# Refined Calibration Model for Improving the Orientation Precision of Electron Backscatter Diffraction Maps

**DOI:** 10.3390/ma13122816

**Published:** 2020-06-23

**Authors:** Aimo Winkelmann, Gert Nolze, Grzegorz Cios, Tomasz Tokarski, Piotr Bała

**Affiliations:** 1 Academic Centre for Materials and Nanotechnology, AGH University of Science and Technology, al. A. Mickiewicza 30, 30-059 Krakow, Poland; ciosu@agh.edu.pl (G.C.); tokarski@agh.edu.pl (T.T.); pbala@agh.edu.pl (P.B.); 2 Department of Physics, SUPA, University of Strathclyde, Glasgow G4 0NG, UK; 3 Federal Institute for Materials, Research and Testing (BAM), Unter den Eichen 87, 12205 Berlin, Germany; gert.nolze@bam.de; 4 TU Bergakademie Freiberg, Institute for Mineralogy, Brennhausgasse 14, 09596 Freiberg, Germany; 5 Faculty of Metals Engineering and Industrial Computer Science, AGH University of Science and Technology, al. A. Mickiewicza 30, 30-059 Krakow, Poland

**Keywords:** scanning electron microscopy, electron backscatter diffraction, Kikuchi diffraction, projection center, orientation precision

## Abstract

For the precise determination of orientations in polycrystalline materials, electron backscatter diffraction (EBSD) requires a consistent calibration of the diffraction geometry in the scanning electron microscope (SEM). In the present paper, the variation of the projection center for the Kikuchi diffraction patterns which are measured by EBSD is calibrated using a projective transformation model for the SEM beam scan positions on the sample. Based on a full pattern matching approach between simulated and experimental Kikuchi patterns, individual projection center estimates are determined on a subgrid of the EBSD map, from which least-square fits to affine and projective transformations can be obtained. Reference measurements on single-crystalline silicon are used to quantify the orientation errors which result from different calibration models for the variation of the projection center.

## 1. Introduction

In materials science, electron backscatter diffraction (EBSD) is a standard tool to obtain spatially resolved crystallographic information from polycrystalline samples in the scanning electron microscope (SEM) [1,2]. EBSD is based on the measurement of Kikuchi diffraction patterns in a gnomonic projection [3] on a planar screen placed near the sample, with the position of the SEM beam on the sample determining the center of the projection relative to the detector plane [4]. Because the knowledge of the gnomonic projection center (PC) is necessary to carry out any angular measurements on the detector screen, a key problem for various diffraction methods in the SEM is the determination of the exact position of the electron beam meeting the sample surface, relative to the detector plane [1,2,4,5,6,7,8,9,10,11,12,13,14,15,16,17,18,19,20,21]. In addition to EBSD, the technique of electron channeling orientation determination (eCHORD) also requires an accurate knowledge of the incident beam geometry for precise orientation determination when large sample areas are scanned [22,23]. The assumption of an incorrect projection center can bias the local crystallographic parameters which are obtained by the analysis of EBSD patterns, and thus has a direct influence on the precision of orientation data from the sample, the lattice parameters, the sensitivity to pseudosymmetry issues, and the quality of phase discrimination, among other issues [20,21,24,25,26,27,28]. In this way, the projection center is of key importance for the principle of the EBSD method, but its quantitative influence on errors of the final EBSD orientation data often is not completely transparent.

In the current paper, we describe the challenges for an accurate treatment of the projection center, using the example case of low-magnification EBSD maps spanning areas of several square millimetres on a silicon sample. In maps of these dimensions, the characteristic systematic changes of the projection center within a map become particularly clear. Using measurements on single crystalline silicon, we analyze the orientation consistency which can be achieved by the use of various models for the position of projection center within the SEM scan. These results on relatively large scale maps can be relevant, for example, to applications of silicon in microelectromechanical systems [29,30], but also in general for materials with large grain sizes, as observed, e.g., in geological materials [31,32]. Because of the high perfection of single crystalline silicon, the magnitude of the inherent orientation variations in the material can be neglected compared to the orientation variations which are caused by the precision and accuracy of the EBSD pattern analysis itself. We assume that the Si material can be described by a fixed, single true orientation, which is unknown, but which can be consistently estimated from the EBSD orientation data. The observed, apparent, misorientations relative to this fixed orientation are considered as an error signal. In order to quantify the precision of the EBSD orientation determination in dependence on the projection center model, we adapt analytical tools which have been developed for the description of small inter- and intragranular misorientations [33,34,35]. We quantify the precision of the EBSD orientation data by the resulting misorientations to the mean orientation in a map, as well as the pairwise misorientation angles as a function of the map point pair distance. These values will indicate how consistently the experimental projection center in different parts of the map is approximated by the calibration models. Moreover, the statistical parameters which we use for a description of the observed misorientation distributions can serve as figures of merit for the precision of specific EBSD setups [36].

## 2. Experimental and Theoretical Details

### 2.1. Relationships between SEM Imaging Parameters and Projection Center

**Figure 1:**
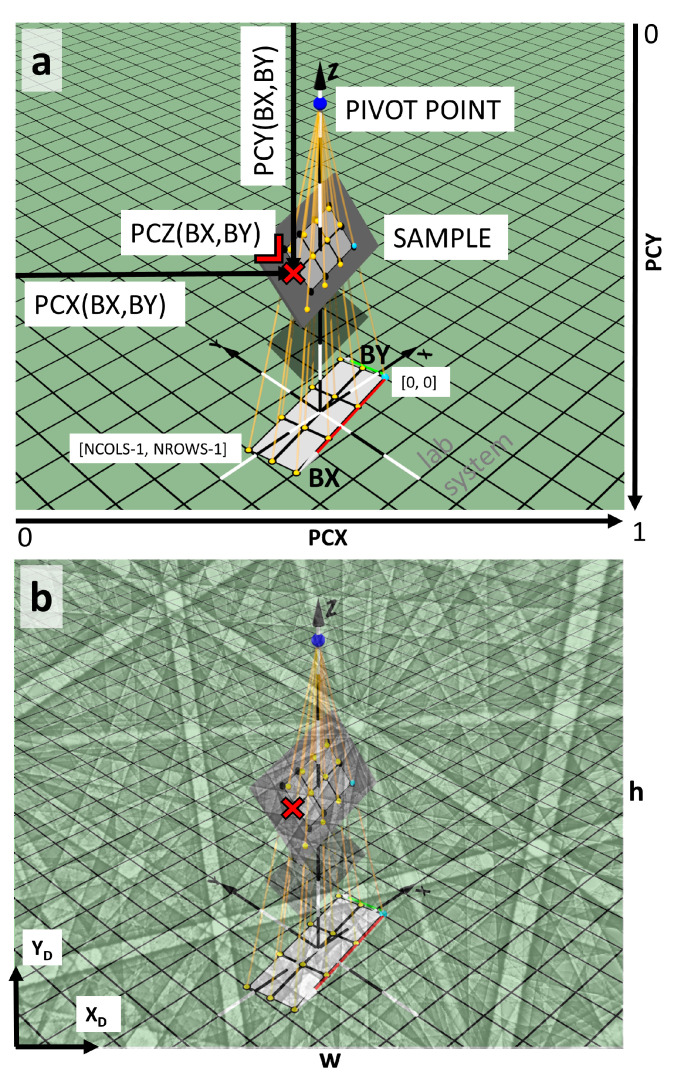
Illustration of the 3D geometry relevant to the EBSD projection center. (**a**) projection geometry of the SEM beam scanning the sample as seen from an EBSD detector screen plane in a general position. The scan pattern shows the combined effects of scan rotation and the sample tilt; (**b**) correspondingly simulated Kikuchi pattern with (PCX,PCY,PCZ)=(0.463,0.516,0.6) as a semi-transparent overlay on the screen plane. The projection center is at the position of the electron beam on the sample, PCZ units of the screen height behind the pattern, measured normal to the screen plane. The decreased visibility of the scan geometry in (**b**) is intended to remind the reader that the scan geometry is usually hidden by the detection screen with the Kikuchi diffraction pattern.

In order to motivate the specification of a mathematical model for the systematic change of the projection center on the sample, we recall the basic geometry of an EBSD experiment in Figure 1. The task of the mathematical model will be to provide the 3D coordinates of the electron beam positions on the sample relative to the detector screen, as a function of the current scan position within the SEM scan (i.e., the column and row of a pixel in the resulting SEM image). In the following, we will use the coordinate system conventions for EBSD as described in [4]. With respect to the example Kikuchi diffraction pattern shown in Figure 1b with the relative projection center coordinates of (PCX,PCY,PCZ)=(0.463,0.516,0.6), this convention means that the 3D geometric coordinates relative to the lower left edge of the phosphor screen are $X_{D}=PCX\;\cdot \;w$, $Y_{D}=\left(1-PCY\right)\;\cdot \;h$, and $Z_{D}=-PCZ\;\cdot \;h$, with the physical screen dimensions of the detector as w= 31,600 μm and h=3w/4 for the full detector area ($e^{-}$Flash${}^{\mathrm{HR}}$, Bruker Nano GmbH, see also below). Thus, the electron beam in Figure 1b is at $X_{D}=$ 14,630.8 μm to the right, $Y_{D}=$ 16,305.6 μm up from the lower left corner of the detection screen, and at $Z_{D}=-$18,960.0 μm in front of the screen (i.e., below the drawing plane).

For the measurement in the SEM, we assume that, in order to scan a grid of image points on a sample, the SEM scan generator deflects the beam from its neutral position into the direction to the current map point, and that the bundle of the deflected beams can be assumed to originate from a common virtual pivot point [37], see Figure 1a. Lower magnifications in the SEM correspond to larger deflection angles of the incident electron beam. Due to the non-parallel directions of the incident beam at different map positions, a regular grid of map points for a horizontal sample will be distorted if the sample is tilted to a general position [12]. In this case, a square on the planar sample without tilt will be transformed into a general quadrilateral on the tilted sample plane. The simplest distortion that can be achieved by a systematic alignment procedure is a trapezoidal distortion, i.e., if the horizontal beam scan is parallel to the tilt axis of the sample [12]. In the general situation, parallel lines of the scan on the untilted sample will not stay parallel in the scan on the tilted sample; but straight lines between scan points remain straight lines in the scan on the sample.

The geometrical calibration of the EBSD measurement is given if we have determined all parameters to specify the physical beam scan positions in relation to the EBSD screen as shown in Figure 1b with a resulting Kikuchi pattern. As we tried to illustrate by this figure, the projection center is not a “point in the Kikuchi pattern”, often called “pattern center”, but the projection center is the location of the beam on the sample surface, measured relative to the detection screen, independent of whether there is a Kikuchi diffraction pattern on the screen or not. This is why we prefer the term “projection center” instead of “pattern center”. The projection center is essential to calibrate the correct measurement of angular distances on the phosphor screen, and these angular measurements can also be applied, for example, to a general electron scattering intensity distribution on the screen irrespective of any diffraction effects [38]. As can be seen in Figure 1a, the treatment of the exact variation of the projection center with the scan position in the map will become more and more relevant as the absolute dimensions of the mapped area become a measurable part of the physical dimensions of the screen, i.e., its physical width *w* and height *h*. Because the dimensions of the commercial EBSD system detector screens are typically in the order of 30 mm, EBSD maps with dimensions of a few mm extension can clearly be expected to show a strong influence, as the diffraction pattern will then move by visually notable distances in the order of 10% of the detection screen. At the other extreme, for very small scan dimensions, a sufficiently accurate projection center position is required for measurements of local lattice parameters, including strain because the accuracy of these values is inherently limited by the assumed projection geometry. For example, in [13], it has been estimated that an uncertainty of 0.005 in the projection center results in uncertainty of about $10^{-3}$ in the strain state, i.e., a variation of 0.1% in lattice parameters.

As a consequence of the influence of the scan size relative to the screen size, various levels of approximations can be made in dependence on the necessary precision of the projection center coordinates. The simplest assumption involves a constant effective projection center for the complete map, which means that we would then neglect an actual variation in the order of $\Delta PC\approx \frac{\Delta x}{w}$, given by the EBSD map width Δx relative to the EBSD screen width *w*. With physical screen sizes *w* of the order of 30 mm, and allowing a maximal variation of the projection center in the order of ΔPC≈0.001 to 0.005 in in the map, for example, we can estimate a maximum map dimension of Δx=ΔPC·w≈(0.001to0.005)·30 mm=30to150 μm which would still meet the requirement of the corresponding PC precision. A similar order of magnitude estimation results when we consider that standard, low resolution EBSD patterns contain roughly 100 pixels on a 30 mm EBSD screen. In this case, we obtain an effective pixel size of 300 μm, which is limiting the magnitude of shifts of pattern features which can actually be detected. For example, if we optimistically postulate a sensitivity to a 1/10th pixel shift in the projection center, this would correspond to a 30 μm shift of the beam. These estimations show that, for high-magnification EBSD maps with dimensions of a few tens of microns, the variation of the projection center in the map can be neglected in many applications. In the limit of experiments aiming at absolute accuracy for local lattice parameters, however, the change of projection center with map point position will always be relevant, independently of the spatial map dimensions.

### 2.2. Projective Transformation Model

The geometrical situation shown in Figure 1a can be described by projective geometry, where the changes of the scan grid for different tilts of the sample can be treated by a projective transformation using homogeneous coordinates [39]. A projective transformation allows for the fact that parallel lines in the scan on the untilted sample will not stay parallel anymore on a tilted sample in the general case. The special case of an affine transformation preserves the parallelism of lines, but still allows for translations, scalings and shears, which can be a sufficient approximation in many cases.

Under these conditions, the relationship between the 2D beam scan indices [BX,BY] and the 3D projection center values [PCX,PCY,PCZ] can be described by a projective transformation P

(1) $$P=\left(\begin{array}{ccc} P_{11} & P_{12} & P_{13}\\ P_{21} & P_{22} & P_{23}\\ P_{31} & P_{32} & P_{33}\\ P_{41} & P_{42} & 1 \end{array}\right)$$

where the matrix P takes the homogeneous column vectors $b={\left(BX,BY,1\right)}^{T}$ to the homogeneous vectors $c\left(BX,BY\right)={\left(PCX,PCY,PCZ,1\right)}^{T}$ via:

(2) $$P\;\cdot \;b=w=w\;\cdot \;c=w\;\cdot \;{\left(PCX,PCY,PCZ,1\right)}^{T}={\left(w_{1},w_{2},w_{3},w_{4}\right)}^{T}$$

As can be seen in Equation (2), the result w of the projective transformation P is not c directly, but c scaled by a real factor *w* which depends on b. The final projection center values can be obtained from w by dehomogenization, i.e., by dividing by the fourth component of w (for $w_{4}\neq 0$):

(3) $$PCX=w_{1}/w_{4}\;PCY=w_{2}/w_{4}\;PCZ=w_{3}/w_{4}$$

A special case results when setting $P_{41}=P_{42}=0$, leading to an affine transformation A

(4) $$A=\left(\begin{array}{ccc} A_{11} & A_{12} & A_{13}\\ A_{21} & A_{22} & A_{23}\\ A_{31} & A_{32} & A_{33}\\ 0 & 0 & 1 \end{array}\right)$$

which leaves the 4th component of the homogeneous projection center vector at w=1:

(5) A·b=c

From observed pairs of beam scan grid indices and projection center vectors, a best-fit affine transformation matrix A can be found by least-squares solution to a linear matrix equation:

(6) $$B_{E}A^{T}=C_{E}$$

where $B_{E}$ and $C_{E}$ are n×3 (n≥3) matrices with rows i=1,…,n of the experimental data points $b_{E}^{\left(i\right)}=\left(BX_{i},BY_{i},1\right)$ and $c_{E}^{\left(i\right)}=\left(PCX_{i},PCY_{i},PCZ_{i},1\right)$, which are the beam scan grid indices and observed projection center values, respectively. To solve the overdetermined matrix Equation (6) for $A^{T}$, we have used the leastsqr outine available in the numpy.linalg Python package [40].

The affine projection center fit has also been used in [20] using a similar pattern matching approach as described here. For the more general projective transformation P, the determination of the transformation matrix from to observed points is a well known problem in computer vision [39]. Various methods for finding the best-fit projective transformation for point sets in 3D (i.e., 4D homogeneous coordinates) are discussed in [41]. For the current application, we have implemented an iterative optimization routine to find a projective matrix P by starting from an affine transformation and minimizing the root mean square deviation to the experimental projection center vector components. We have found this approach to work well when $P_{41}$ and $P_{42}$ are very small compared to all other elements and the effect of P remains close to an affine transformation.

### 2.3. Kikuchi Pattern Matching for Projection Center Calibration

In order to obtain the experimental estimations $c_{E}^{\left(i\right)}=\left(PCX_{i},PCY_{i},PCZ_{i},1\right)$ of the PC coordinates from the experimental Kikuchi patterns, individual values for the orientation and the projection center $c_{E}^{\left(i\right)}$ of a map point *i* where determined by a best-fit pattern matching approach as described in [28,42]. The best fit parameters were determined at the maximum of the normalized cross correlation coefficient (NCC) between the simulated pattern and the experimental pattern. In comparison to other available measures used for Kikuchi pattern matching, the NCC is very robust to changes in brightness and contrast in the analyzed Kikuchi patterns, among other useful properties of the NCC [43,44,45,46]. The pattern matching procedure compares background-corrected Kikuchi patterns which have been normalized to a mean of zero and unit standard deviation [47]. As starting values for the optimization procedure, we used the values of the local orientation and PC as given by the EBSD system software (Bruker Esprit 1.94, Bruker Nano GmbH, Berlin, Germany). The starting values which were optimized using the Nelder-Mead (NM) algorithm as implemented in the NLOPT nonlinear optimization package [48]. In order to test the convergence, the NM optimization for each map point was restarted several times with different initial values changed by random deviations, i.e., orientations within one degree misorientation angle, and the PC values changed in a range of ±0.02. We found that a number of five restarts were sufficient in most cases. In the present study, we used experimental Kikuchi patterns with a resolution of 400×300 pixels, which were resampled to a resolution of 800×600 pixels to reduce possible discretization effects in the projection center optimization process.

In a first step, the optimization procedure varied the orientation parameters and the projection center coordinates on a 7×7 subset of points covering the complete map. Taking the NCC as a quality measure, outliers to be discarded can be defined as data points with a NCC below a certain threshold. The resulting values for the projection coordinates where then fitted to an affine and a projective transformation as discussed above. A consistent calibration of the projection center for all points in the map is finally given by the nine parameters of the affine projection matrix A of Equation (4) and the 11 parameters of the projective projection matrix P of Equation (1), respectively.

After the projection center calibration matrices A and P have been determined in the first step, we subsequently optimized the orientation values for all points in the map, fixing the local projection center coordinates at the scan points [BX,BY] as given by the calibration matrices. Only the orientation parameters (Euler angles) were varied in this final optimization step. On a single-crystalline sample without any internal orientation changes in the material, an idealized analysis should result in the actual orientation of the material, which would be identical for all data points in the map of the single crystal. The limited precision and accuracy of the complete EBSD data analysis pipeline, however, will result in systematic and random errors of the actual experimental orientation data, even from a nearly perfect material. If we can expect that the orientations vary randomly around a mean orientation, we can take this mean orientation as the reference orientation. While we cannot conclude on a possible systematic deviation of this mean orientation data from the true orientation of the material, we can analyze the precision of the projection center calibration by the spread in the final, optimized orientations relative to the mean orientation. We can expect that an improved precision of the projection geometry should lead to less spread of the orientation data around the average orientation in the EBSD map of a single-crystalline material.

### 2.4. EBSD Measurements

The EBSD measurements have been performed using a field-emission gun scanning electron microscope (FEG-SEM) LEO 1530VP (Zeiss, Oberkochen, Germany). Using the high-current mode and a 120 μm aperture, the Shottky field emitter delivered a probe current of about 10, …, 12 nA in high vacuum mode.

For the EBSD-pattern acquisition an $e^{-}$Flash${}^{\mathrm{HR}}$ (Bruker Nano GmbH, Berlin, Germany, nominal CCD-chip resolution: 1600 × 1200 pixels) was used. The analyzed and stored raw patterns were 8-bit gray-scale images with a size of 400 × 300 pixels. The dwell times per pattern were 15 ms. We did not apply any frame averaging. For the EBSD orientation data analysis, we applied the internal EBSD system software (Esprit 1.94), as well as the MTEX toolbox [49].

The sample used in this study was obtained from a commercial Si(001) wafer as delivered, without any further sample preparation. The sample was deliberately mounted in a slightly tilted position relative to the surface of the sample holder, in order to include the effect of a general tilt in the projection center calibration. The Si sample was imaged at low magnification (nominally 85×) with 100 × 75 map points at a nominal 40 μm step size, leading to a total mapped area of 4 mm × 3 mm extension.

## 3. Results

### 3.1. Projection Center Calibration

**Figure 2:**
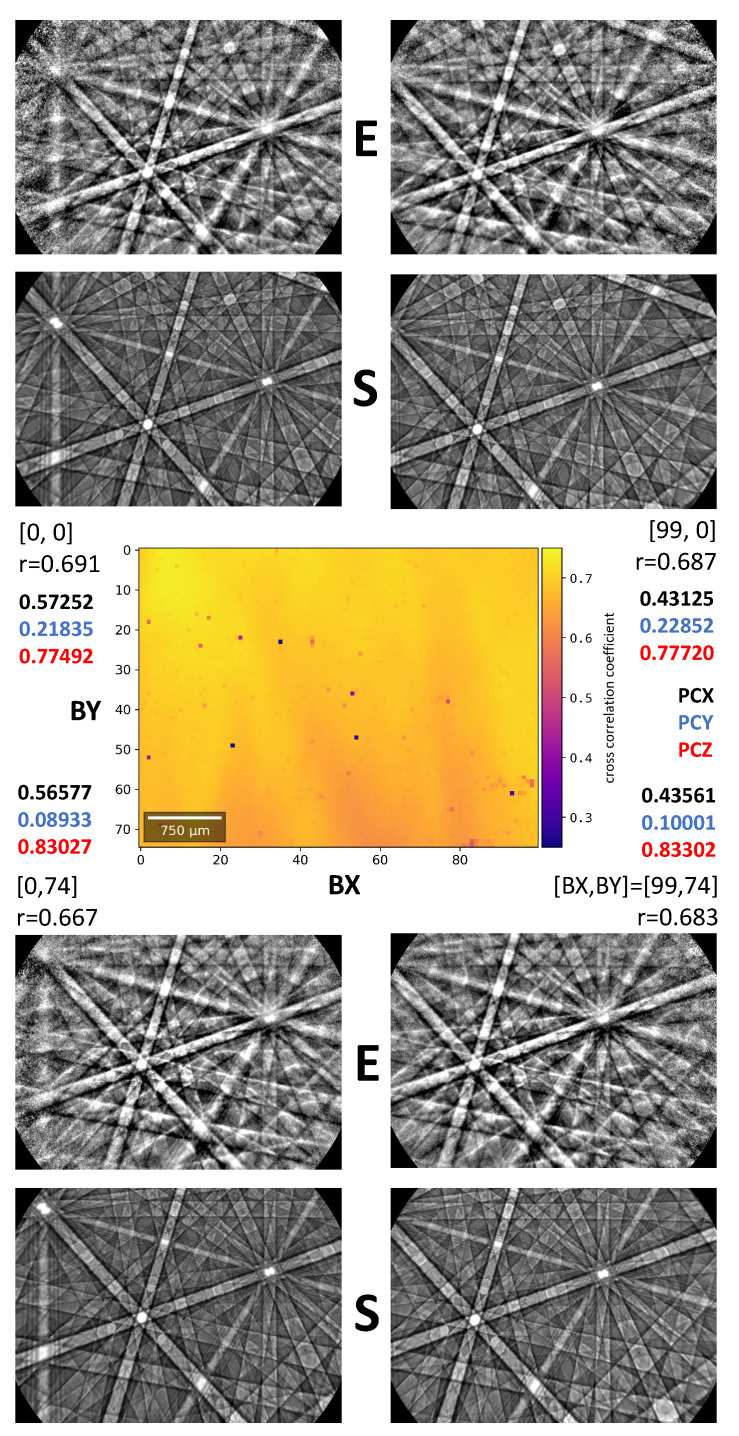
Projection center (PC) pattern matching results for the corner points of the map. The experimental patterns are marked by “E” and the corresponding best-fit simulations are shown by “S”. The best-fit PC values PCX,PZY,PCZ are shown in color, below the beam scan indices [BX,BY] and the value of the normalized cross-correlation coefficient *r*. In agreement with the geometry of the scan relative to the EBSD screen [4], the Kikuchi patterns can be seen to move to the left with increasing BX, and upwards with increasing BY. Because the vertical movement with increasing BY also moves the beam away from the screen on the tilted sample, increasing PCZ, the patterns in the lower row also appear magnified, as can be seen by the slightly broader Kikuchi bands. The full map of *r*-values resulting during the final orientation refinement shown in the center.

For the calibration of the projection center in the map, we carried out the pattern matching procedure as described above for a subset of 7×7 grid points across the map. In Figure 2, we show the results for the four corner points of the scan having a total of 100 columns and 75 rows. The beam scan indices [BX,BY] correspond to the column BX and row BY in the scan. The overall level of agreement between simulations and experiment can be seen by the Kikuchi patterns, marked “S” (simulation) and “E” (experiment), respectively. The pattern similarity is quantified by the normalized cross correlation coefficient *r*, which is mainly in the range 0.6<r<0.7 for the current sample. In the center of Figure 2, we show the full *r*-map which resulted after the orientation refinement as will be discussed below. Dark spots indicate positions of lower quality Kikuchi patterns due to contamination on the sample surface. The best-fit projection center coordinates PCX,PZY,PCZ at the example points are shown in color in Figure 2. In agreement with the geometry of the scan relative to the EBSD screen [4], the Kikuchi patterns can be seen to move to the left with increasing BX, and upwards with increasing BY. Because the vertical movement with increasing BY also moves the beam away from the screen on the tilted sample, this also leads to an increasing PCZ, and the patterns in the lower row also appear zoomed-in, as is visible by the slightly broader Kikuchi bands. The key topic of the present paper is to quantify the existing effect of this observed movement of the Kikuchi patterns on the resulting orientation data. While the imaged Kikuchi patterns change due to the movement of the electron emission spot relative to the screen, the orientations at these positions on the silicon sample remain essentially constant. The consistency of the resulting EBSD orientation data can be judged by how well the constraint of an identical orientation is fulfilled on the mapped positions. Based on Figure 2, it can be supposed that the measurable variations would be largest if the resulting orientations from the corner regions of the map are compared relative to each other because then also the relative changes in the Kikuchi patterns are largest.

**Figure 3:**
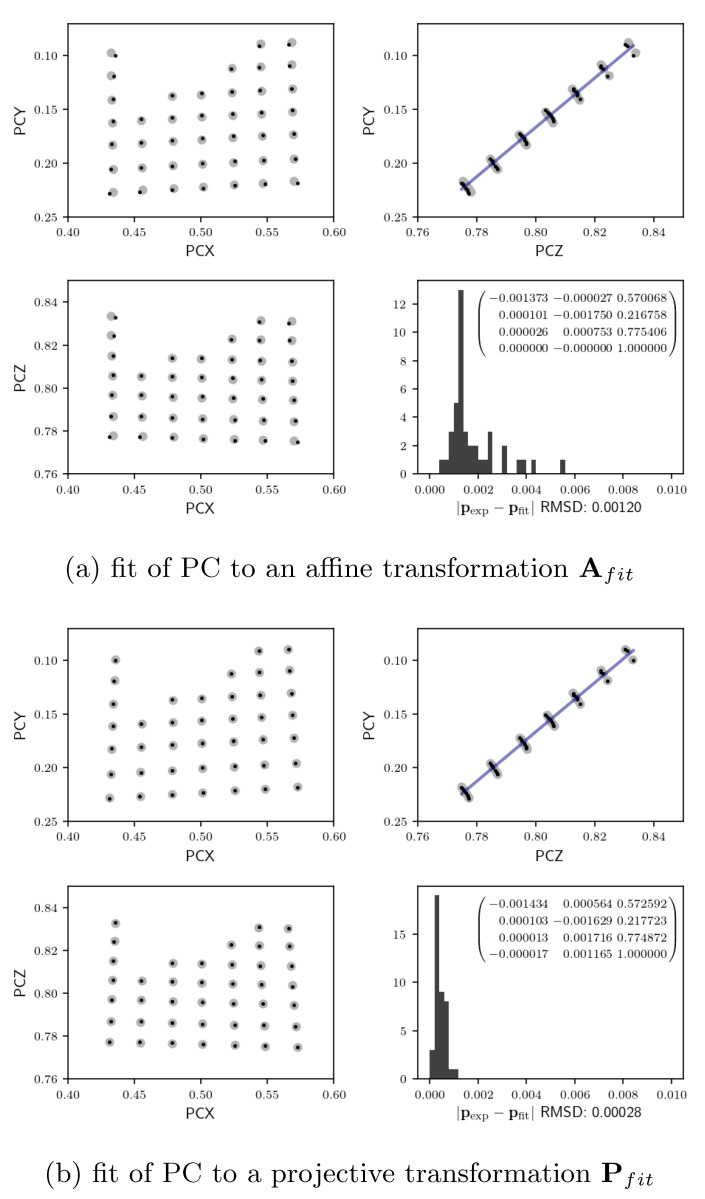
Fit of transformation matrices to the individual experimental projection center (PC) coordinates. The small black points are the experimental data points, and the larger gray filled circles are the respective fit results according to (a) the affine transformation matrix $A_{fit}$ and (b) the projective transformation matrix $P_{fit}$ shown in the lower right panels. Experimental data points were measured on a 7×7 subgrid and selected according to a minimum allowed cross-correlation coefficient $r_{min}=0.65$. This resulted in the 41 calibration points as shown in the figure.

The set of 7×7 calibration points resulted in pairs of the experimental data points $b_{E}^{\left(i\right)}=\left(BX_{i},BY_{i},1\right)$ and $c_{E}^{\left(i\right)}=\left(PCX_{i},PCY_{i},PCZ_{i},1\right)$ with i=1,…,49. Using a threshold value of $r_{min}=0.65$, the experimental data points were fitted to the affine and projective transformations as described above. The results are shown in Figure 3. In the lower right panels of (a) and (b), we show the histograms of the absolute difference between the experimental and the fitted projection center vectors, and the respective transformation matrices. For calculation of the error values shown in the histograms in Figure 3, we scaled PCX to also correspond to the image height, in order to measure all values relative to the same reference length. The resulting root mean square deviation (RMSD) between experiment and simulation is 0.00120 for the fit of an affine transformation, and 0.00028 for the fit to the projective transformation model, indicating an increase of the error by a factor larger than 4 for the affine model. Taking the absolute height of the EBSD detector area for the image as 23.7 mm, the RMSD values would correspond to 28.4 μm for the affine model and 6.6 μm for the projective model, which illustrates the size of the systematic error introduced by a limitation to an affine transformation. The consistently better fit of the projective model can also be noticed in the panels which show 2D maps of pairs of the three projection center coordinates. The missing nine grid points are ouliers as defined as having an NCC less than the minimum value $r_{min}=0.65$. The projective transformation takes into account that the scan grid lines on a tilted sample are not parallel anymore. In contrast, the affine transformation model conserves parallelism and can only treat scalings and shears. The map of PCY vs. PCZ should be a straight line if the sample would have rotated only around the *x*-axis, and from the slope of the we can estimate the corresponding sample tilt relative to the detector. The estimation using linear regression leads to a tilt angle of $\vartheta_{x}^{LR}=23.12^{\circ }$ of the sample plane normal in the detector (y,z)-plane. Taking into account a possible small tilt of the EBSD detector, as well as of the sample mount, the value of $\vartheta_{x}^{LR}$ is compatible with the nominal 70${}^{\circ }$ sample tilt, which is measured relative to the microscope *z*-axis. In Figure 3, we see a very systematic deviation of the experimental data from the line in the relationship between PCY and PCZ. This is due to an additional small tilt of the sample plane, which makes the sample plane normal to point slightly away from the (y,z)-plane, i.e., the sample plane normal is not at a right angle relative to the *x*-axis of the detection screen.

The resulting transformation matrices allow for describing the general orientation of the sample plane relative to detector. This can be achieved by calculating the images of the points [0,0,1], [1,0,1], and [0,1,1], which span the resulting scanning plane. We measure the orientation of the scanning plane in the detector system [4], for a sample normal reference direction along the positive *z*-axis (i.e., the detector screen normal pointing away from the sample). For the experimental data above, we find that the normal vector of the scanning plane is rotated by $\vartheta_{x}=$ 23.24${}^{\circ }$ from the (z,x)-plane (towards positive *y*) and by an angle $\tau_{y}=-$2.16${}^{\circ }$ relative to the (y,z)-plane (towards negative *x*). Both the affine and the projective transformation model give the same sample plane normal direction within the precision stated above. This observation is consistent with the fact that both types of transformations conserve the co-planarity of the scan points. The difference of the two transformation models is mainly on the position of the map points within the sample plane, i.e., the projective transformation allows parallel lines in the beam scan grid to become non-parallel within the sample plane.

### 3.2. Orientation Analysis

The effect of the different calibrations of the projection center in the map can be seen by an analysis of the resulting orientation data. This allows us to quantify the influence of the observed movement of the Kikuchi patterns on the orientation data from nearby map points (the local orientation precision), as well as on the changes of orientations of map points as a function of their mutual separation (global orientation precision).

#### 3.2.1. Local Orientation Precision

**Figure 4:**
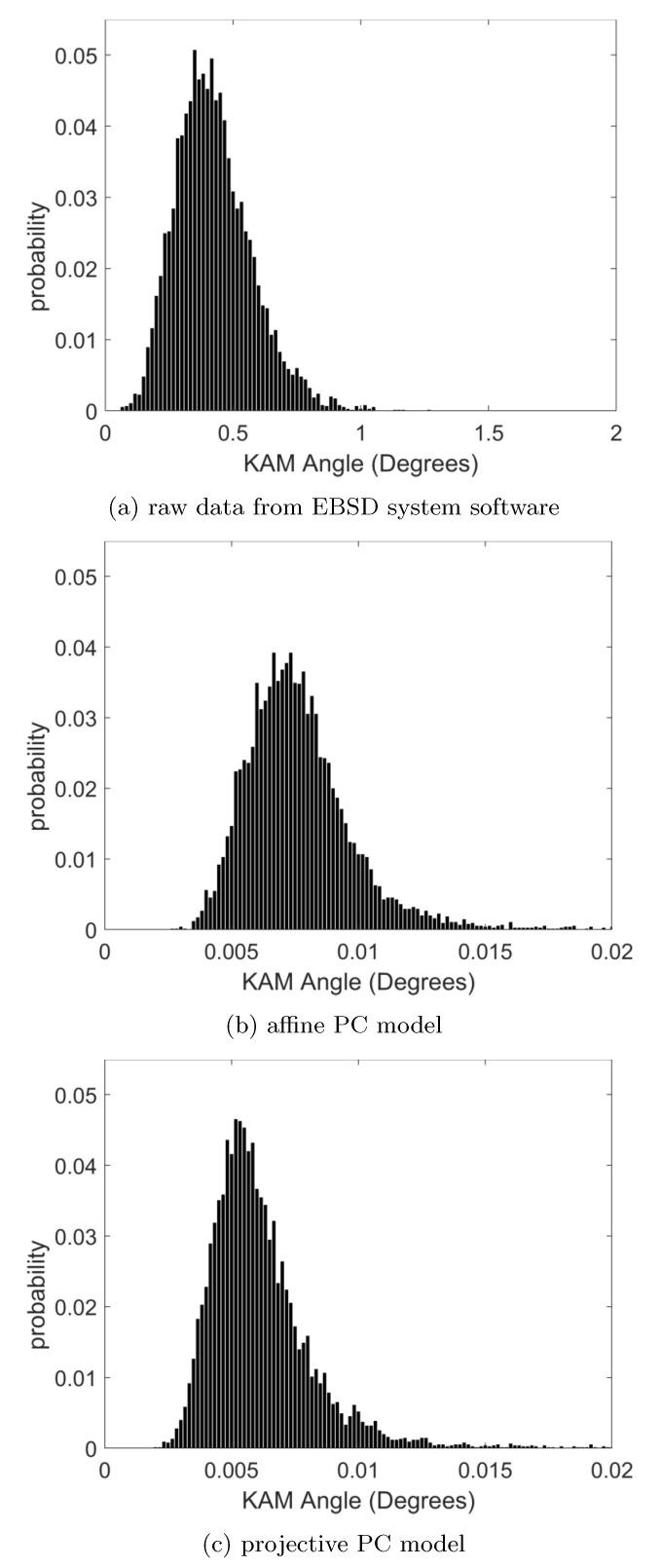
Histograms of the Kernel Average Misorientation angle for the orientation maps resulting from different analysis approaches. (a) raw data from EBSD system, (b) refined orientations using affine PC calibration, (c) refined orientations using projective PC calibration. Note that the *x*-axis limit in the top panel (a) is larger by a factor of 100 compared to the other two panels.

The local variation in the orientation data can be quantified by misorientation values in the neighborhood of each map point [50]. In Figure 4, we show the histograms of the Kernel Average Misorientation angle (KAM) to the nearest neighbor in the orientation maps (calculated using the first order KAM angles as implemented in MTEX 5.2.8 [51]). The initial values which were obtained using the orientation from the EBSD system software are shown in Figure 4a, compared to the KAM values obtained from the refined orientation map using the affine transformation model in Figure 4b and the projective transformation model in Figure 4c. Qualitatively, we can see that the pattern matching based values in (b) and (c) are almost two orders of magnitude lower than the result in (a), noting that the angular scale goes up to 2 degrees in (a) compared to the maximum range of 0.02 in (b) and (c). The distribution of the KAM angles in the histograms are summarized by the median and 95th percentile values given in Table 1. While the projective transformation model results in marginally lower KAM values than the affine transformation model, the most significant difference is seen relative to the raw orientation data that was provided by of the EBSD system software. Comparable improvements of the KAM values have been shown previously for measurements on Si samples and map areas of about 1 mm${}^{2}$ in [20]. We note that different types of EBSD systems using various data analysis approaches can be expected to provide the raw orientation data with their different typical precision [52,53,54,55].

Because the variation of the projection center position is small from one map point to its neighbor, the local KAM values do not show a strong dependence of the spatial position in the map. The error of the local projection center position will be approximately constant for neighboring data points and thus the resulting orientations and the corresponding local KAM do not change very much locally. We have observed that the only systematic features are at the contamination spots, and this is why the KAM maps are not shown explicitly. The KAM histograms in Figure 4 convey the key information.

#### 3.2.2. Global Orientation Precision

The EBSD orientation data in the present study can effectively be considered to originate from a single large grain of nearly perfect Si. This makes it possible to apply some well-known characterization concepts for intragranular orientation variations in materials to describe the apparent, spatially dependent orientation variations which are induced purely by an inaccurate assumption for the projection center in the EBSD data analysis.

**Figure 5:**
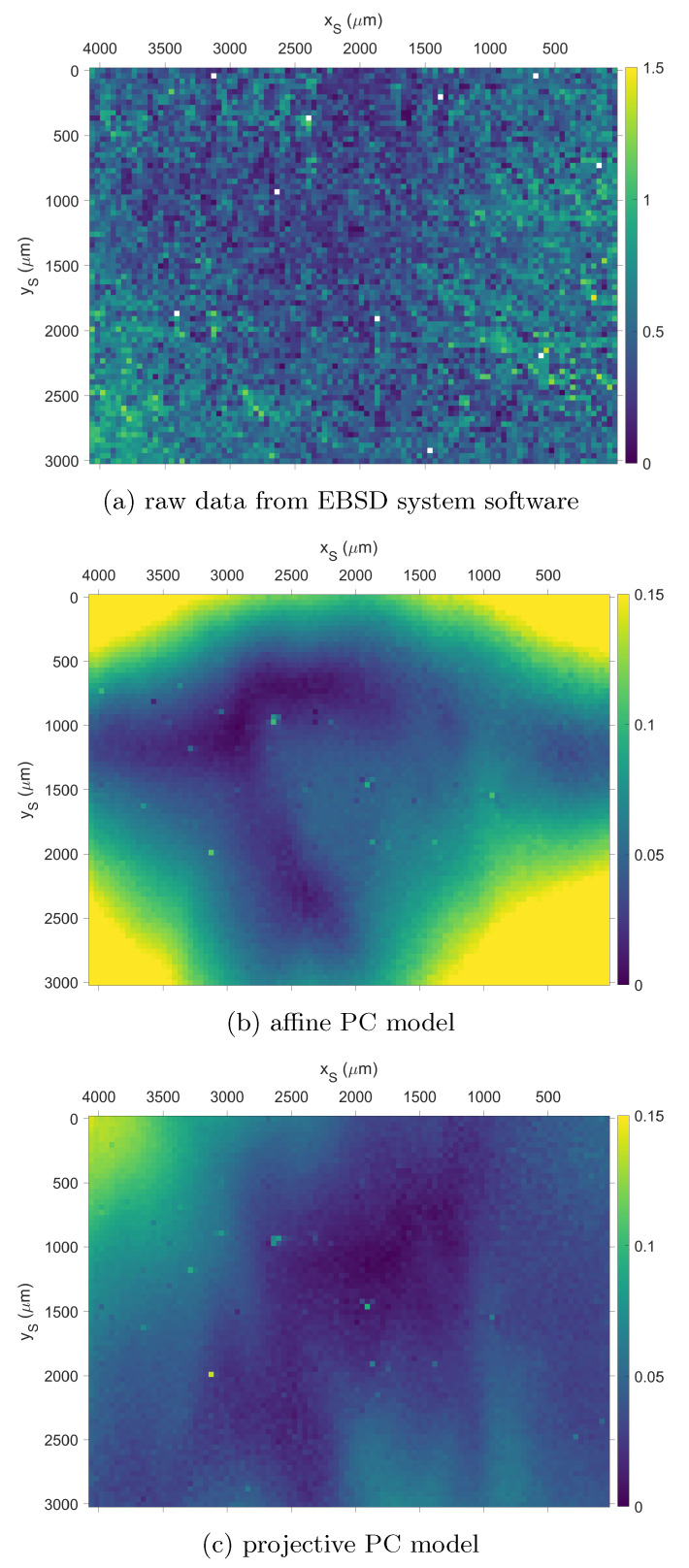
Maps of the misorientation angle to the mean orientation. (a) raw data from EBSD system, (b) refined orientations using affine PC calibration, (c) refined orientations using projective PC calibration. Scale in degrees, note that the scale for the raw data in (**a**) is increased by a factor of 10.

A useful measure to illustrate the variation of orientations in the map is the misorientation angle relative to the mean orientation [34,35,49,56,57]. In Figure 5, we show the maps of the misorientation angle relative to the mean orientation, which result from using the different calibration approaches for the projection center. As we can see by a comparison of the angular scale in Figure 5a to the scales in (b) and (c), the pattern matching orientation refinement in (b,c) reduces the overall, apparent, orientation variation in the map by about an order of magnitude. The comparison of the affine model applied in (b) to the results from the projective model in (c) shows that the projective model leads to a significantly reduced spatial orientation deviation, with the largest difference remaining in the upper left corner of the map. This improvement shows the signifance of the projective calibration in terms of the total orientation consistency in the map. In comparison, the local KAM values are not influenced as critically by the choice of an affine or projective model, as we have seen in Figure 4.

The maps in Figure 5 can be quantitatively described by an analysis of the components of the rotation vector relative to the mean orientation. The corresponding rotation vectors $\vec{\Theta }$ are defined to point into the direction of the rotation axis and have a length corresponding to the rotation angle (see e.g., Equation (2.51) and Chapter 3 in [58]). The advantage of the rotation vector is that it linearizes the space of rotations for small rotations, using the components $\Theta_{x},\Theta_{y},\Theta_{z}$ as coordinates, i.e., see the discussion of the Lie group SO(3) in [58]. This makes it possible, for example, to analyze small random rotations in terms of Gaussian distributions for $\Theta_{x},\Theta_{y},\Theta_{z}$ (note that this is not valid for a rotation description by Euler angles). In the context of EBSD, the rotation vector and closely related quantities have been used before to analyze plastic deformations relative to the mean orientation in individual grains [34,56,59,60].

**Figure 6:**
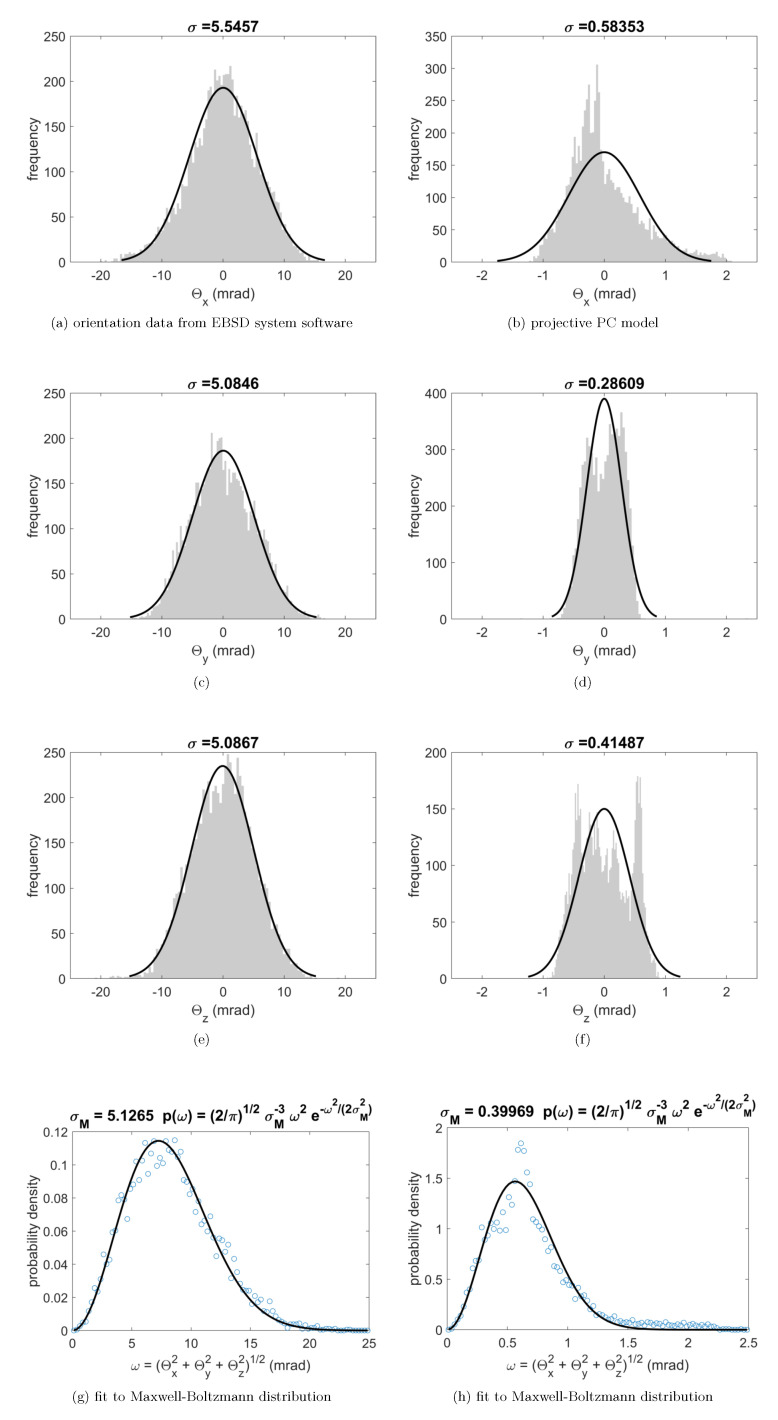
(a–f) Histograms of the rotation vector components of the misorientation relative to the mean orientation, (a,b) $\Theta_{x}$, (c,d) $\Theta_{y}$, (e,f) $\Theta_{z}$; and (g,h) the histograms of the misorientation angles ω. The black solid lines correspond to fits of Gaussian distributions (top three rows) and Maxwell–Boltzmann distributions (lower row) with the standard deviations shown on top of each plot. The left column shows the data for the raw EBSD maps, the right column shows the results from the orientation data which was refined by the projective model. Note that the angular scale for the left column is ten times larger than for the right column.

For the measurement of the Si sample, the histograms of the rotation vector components $\Theta_{x},\Theta_{y},\Theta_{z}$ and of the resulting absolute value of the misorientation angle $\omega ={\left(\Theta_{x}^{2}+\Theta_{y}^{2}+\Theta_{z}^{2}\right)}^{1/2}$ are shown in Figure 6. On the left side, in panels (a,c,e), we show the histograms resulting for the orientations from the EBSD system software. It can be seen that the histograms of the rotation vector components for the raw data are very well described by Gaussian distributions with a mean of zero and a standard deviation $\sigma^{raw}\approx 5\;\mathrm{mrad}$, which are shown as black lines. On the right side, in panels (b,d,f), the rotation vector components are shown which result from orientation refinement using full pattern matching with the projective calibration. These distributions have a spread which is about an order of magnitude smaller than the raw EBSD data, as can be seen by the standard deviations near $\sigma^{proj}\approx 0.4\;\mathrm{mrad}$ around a mean value near zero. The shape of the refined distributions deviates from a Gaussian shape more than the EBSD raw data, and the spread is slightly different for the $\Theta_{x},\Theta_{y},\Theta_{z}$ components. We assign the detailed shape of these distributions to a remaining influence of discretization effects on the pattern fit, which depend on the specific orientation of the Si sample, the resolution of the measured patterns and the solid angle of the captured diffraction patterns [61], in addition to the limits of the theoretical approach itself which neglects or approximates a range of experimentally observable pattern details. These details include the excess-deficiency effects [62] and higher-order Laue zone rings [63], which require numerically more expensive simulations [64].

In the bottom row of Figure 6, we show that the histograms of the misorientation angle ω can be fitted by a Maxwell-Boltzmann distribution, which is the theoretical distribution of the square root of a sum of three random variables which are independently distributed according to Gaussian distributions with mean value zero and the same standard deviation $\sigma_{M}$:

(7) $$p\left(\omega \right)=\sqrt{\frac{2}{\pi }}\frac{\omega^{2}}{\sigma_{M}^{3}}\exp\left(-\frac{\omega^{2}}{2\sigma_{M}^{2}}\right)$$

The Maxwell-Boltzmann distribution is also useful as the limiting case of a general class of distributions used for the statistical description of rotations [65]. If the rotation vector components cannot be assumed to be normally distributed with approximately the same standard deviation and mean zero, other distributions for the misorientation angle $\omega ={\left(\Theta_{x}^{2}+\Theta_{y}^{2}+\Theta_{z}^{2}\right)}^{1/2}$ will result. For example, if the misorientations are effectively two-dimensional, a Rayleigh distribution [34] is obtained.

Applying a least square fit to the Maxwell-Boltzmann distribution in Equation (7), we find $\sigma_{M}^{raw}=5.1$ mrad for the raw EBSD system orientation data, while the refined orientation data shows a value of $\sigma_{M}^{proj}=0.40$ mrad. With respect to the differences which are seen between the orientation maps of the affine model and the projective model in Figure 5, we find an increased value of $\sigma_{M}^{aff}=0.69$ mrad for the affine model using the same analysis. This corresponds to an increase in the spread of orientations $\sigma_{M}$ by a factor of 1.7 between the projective and the affine calibration, and an increase of a factor of 12.7 for the raw EBSD system data.

As can be seen in Equation (7), the Maxwell–Boltzmann distribution depends only on one parameter, namely $\sigma_{M}$, which, in our application, can be seen to approximately describe an effective standard deviation of the components of the misorientation vectors relative to a true mean orientation. Because of this straightforward statististical meaning, the value of $\sigma_{M}$ could be used as a key parameter to describe the orientation precision of a specific EBSD setups. We can expect that $\sigma_{M}$ will depend on the specific combination of SEM, the EBSD system hardware, and the data analysis software, when carried out on easily available Si samples measured in given reference orientations [36].

**Figure 7:**
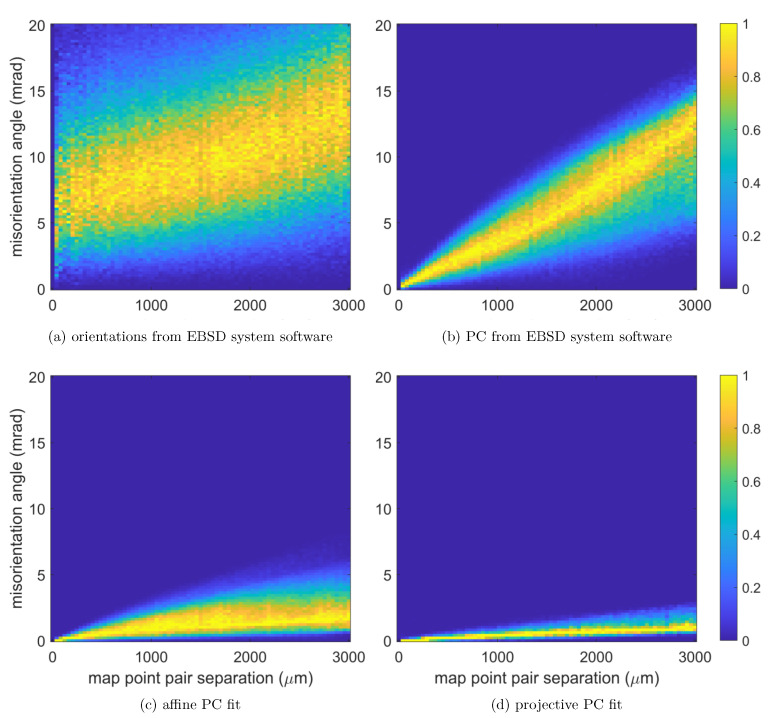
Distributions of the relative misorientation angle between pairs of map points, as a function of the map point pair separation for different projection center models. (a) raw orientations as given by the EBSD system software, (b) refined orientations by pattern matching using the PC calibration of the EBSD system software, (c) refined orientations using the affine PC calibration, (d) refined orientations using the projective PC calibration. The distributions are normalized to the maximum value of the misorientation angle distribution at each distance (bin width 40 μm).

In general, the spread of orientations described by $\sigma_{M}$ will also depend on the spatial distance of the map points which have been used to calculate the distribution of the misorientation angles ω. It can be speculated that pairs of map points which are separated by large distances will also tend to show larger misorientations. We have quantified this expectation by calculating the pairwise misorientation angles for a given number of randomly selected pairs from the map, and counting the number of pairs within specific intervals of distances and misorientation angles as two-dimensional histograms. The resulting two-dimensional histograms for $10^{6}$ pairs from the Si map are shown in Figure 7. In these figures, the distributions of the misorientation angle in each given bin of map point pair separation are normalized to the maximum of the counts for all angles in that bin. In this way, the distance-dependend shift of the maximum and the changes in the width of the distribution is emphasized. The spatial step size in the histogram is 40 μm. For the raw EBSD data, it can be seen that the dominating misorientation values are between 5 and 10 mrad for map point separations in the order of 100 μm, and this interval rises towards values between 10 and 15 mrad for a separation near 3 mm. As can be seen in panel (b), the orientation precision of nearby points can be improved by pattern matching using an unrefined projection center as given by the EBSD system software, but the misorientation at a large distance still tends to similar values as in the raw data. For the affine PC model shown in (c), the misorientation angles stay below 5 mrad, and in (d) we can see that the projective model has a still sharper distribution with angles predominantly below 1 mrad deviation. Thus, the projective model for the projection center also leads to the least increase in relative misorientation between spatially separated data points which nominally have the same orientation. We note that this type of information about the spatially changing, systematic orientation errors is usually not accessible in measurements from polycrystalline maps because the separated points then are very likely to be in different grains. In that situation, the locally changing systematic orientation error is masked by the locally changing grain orientations.

## 4. Conclusions

We have demonstrated the use of a projective transformation model for the calibration of the projection center in EBSD maps of large spatial dimensions, involving low magnifaction modes in the scanning electron microscope.

Using measurements on single-crystalline Si samples, we have shown that, compared to an affine transformation, the projective transformation model leads to a decreased spread of misorientation angles relative to the mean orientation when the orientations are refined using a full pattern matching approach.

For the combination of EBSD system and SEM which was used in the present study, this approach leads to an improvement of the local orientation precision by of about two orders of magnitude (as seen by the KAM maps), and to an improvement in global orientation precision by one order of magnitude (as seen by the misorientation angles relative to the mean orientation).

Ultimately, the approach of fitting the projection center to simulated Kikuchi patterns is limited by the ability of the simulation to take into account all relevant experimental effects. For example, the excess-deficiency effects have not been included in the current simulation model [62]. As has been discussed above, the projection center is essentially related to the purely geometrical relationships in the measurement setup. In order to analyze the absolute precision of the projection center, alternative determinations of the projection geometry could use methods which are independent of the observation of any Kikuchi patterns from the sample, such as shadow casting methods [6,15] or single-crystalline detectors like those discussed in [19].

The problem of the dependence of the projection center on the position within the beam scan is closely related to the observed spatial distortions of EBSD maps when mapping large sample areas by assembling a number of smaller sub-maps into a larger mosaic. As we have shown, the projective transformation model can provide an improved description of the scan geometry in larger EBSD maps, which could help to reduce the number of maps which would need to be stitched together for a given sample area. The projective transformation model is also relevant to SEM in-situ testing setups for mechanical properties of materials, where deformations are measured on large sample areas [66,67]. A precise correction of the spatial distortions will also require the treatment of the electron lens distortions which lead to a deviation from the purely projective distortion model. Methods to correct for these additional influences are well known for optical systems in computer vision [39], and could be implemented by transforming the initial scan incides [BX,BY] according to the radial and tangential disortions caused by the electron optics [68]. The investigation of such additional corrections in the future is also expected to further improve the orientation consistency in EBSD maps.

## Figures and Tables

**Table 1:** Characteristic values of the histograms ot the Kernel Average Misorientation Angle shown in Figure 4.

Orientation Data	KAM Median	KAM 95th Percentile
Esprit 1.94	0.4100${}^{\circ }$	0.690${}^{\circ }$
Affine PC	0.0074${}^{\circ }$	0.011${}^{\circ }$
Projective PC	0.0058${}^{\circ }$	0.010${}^{\circ }$

## References

[1] Schwartz A.J., Kumar M., Adams B.L. (2000). Electron Backscatter Diffraction in Materials Science.

[2] Engler O., Randle V. (2009). Introduction to Texture Analysis: Macrotexture, Microtexture, and Orientation Mapping.

[3] Palache C. (1920). The Gnomonic Projection. Am. Mineral..

[4] Britton T., Jiang J., Guo Y., Vilalta-Clemente A., Wallis D., Hansen L., Winkelmann A., Wilkinson A. (2016). Tutorial: Crystal orientations and EBSD—Or which way is up?. Mater. Charact..

[5] Venables J.A., Bin-Jaya R. (1977). Accurate microcrystallography using electron back-scattering patterns. Philos. Mag..

[6] Biggin S., Dingley D.J. (1977). A general method for locating the X-ray source point in Kossel diffraction. J. Appl. Crystallogr..

[7] Dingley D.J., Longden M., Weinbren J., Alderman J. (1987). Online Analysis of Electron Back Scatter Diffraction Patterns. 1. Texture Analysis of Zone Refined Polysilicon. Scanning Microsc..

[8] Baba-Kishi K.Z. (1998). Measurement of crystal parameters on backscatter Kikuchi diffraction patterns. Scanning.

[9] Krieger Lassen N.C., Bilde-Sørensen J.B. (1993). Calibration of an electron back-scattering pattern set-up. J. Microsc..

[10] Krieger Lassen N.C. (1999). Source point calibration from an arbitrary electron backscattering pattern. J. Microsc..

[11] Carpenter D.A., Pugh J.L., Richardson G.D., Mooney L.R. (2007). Determination of pattern centre in EBSD using the moving-screen technique. J. Microsc..

[12] Nolze G. (2007). Image distortions in SEM and their influences on EBSD measurements. Ultramicroscopy.

[13] Britton T.B., Maurice C., Fortunier R., Driver J.H., Day A.P., Meaden G., Dingley D.J., Mingard K., Wilkinson A.J. (2010). Factors affecting the accuracy of high resolution electron backscatter diffraction when using simulated patterns. Ultramicroscopy.

[14] Maurice C., Dzieciol K., Fortunier R. (2011). A method for accurate localisation of EBSD pattern centres. Ultramicroscopy.

[15] Mingard K., Day A., Maurice C., Quested P. (2011). Towards high accuracy calibration of electron backscatter diffraction systems. Ultramicroscopy.

[16] Basinger J., Fullwood D., Kacher J., Adams B. (2011). Pattern center determination in electron backscatter diffraction microscopy. Microsc. Microanal..

[17] Alkorta J. (2013). Limits of simulation based high resolution EBSD. Ultramicroscopy.

[18] Ram F., Zaefferer S., Raabe D. (2014). Kikuchi bandlet method for the accurate deconvolution and localization of Kikuchi bands in Kikuchi diffraction patterns. J. Appl. Cryst..

[19] Vespucci S., Naresh-Kumar G., Trager-Cowan C., Mingard K.P., Maneuski D., O’Shea V., Winkelmann A. (2017). Diffractive triangulation of radiative point sources. Appl. Phys. Lett..

[20] Friedrich T., Bochmann A., Dinger J., Teichert S. (2018). Application of the pattern matching approach for EBSD calibration and orientation mapping, utilising dynamical EBSP simulations. Ultramicroscopy.

[21] Pang E.L., Larsen P.M., Schuh C.A. (2020). Global optimization for accurate determination of EBSD pattern centers. Ultramicroscopy.

[22] Lafond C., Douillard T., Cazottes S., Steyer P., Langlois C. (2018). Electron CHanneling ORientation Determination (eCHORD): An original approach to crystalline orientation mapping. Ultramicroscopy.

[23] Lafond C., Douillard T., Cazottes S., Graef M.D., Steyer P., Langlois C. (2020). Towards large scale orientation mapping using the eCHORD method. Ultramicroscopy.

[24] Nolze G., Winkelmann A. (2014). Exploring structural similarities between crystal phases using EBSD pattern comparison. Cryst. Res. Technol..

[25] Winkelmann A., Nolze G. (2015). Point-group sensitive orientation mapping of non-centrosymmetric crystals. Appl. Phys. Lett..

[26] Chen Y.H., Park S.U., Wei D., Newstadt G., Jackson M.A., Simmons J.P., De Graef M., Hero A.O. (2015). A Dictionary Approach to Electron Backscatter Diffraction Indexing. Microsc. Microanal..

[27] Hielscher R., Bartel F., Britton T.B. (2019). Gazing at crystal balls: Electron backscatter diffraction pattern analysis and cross correlation on the sphere. Ultramicroscopy.

[28] Winkelmann A., Jablon B.M., Tong V.S., Trager-Cowan C., Mingard K.P. (2020). Improving EBSD precision by orientation refinement with full pattern matching. J. Microsc..

[29] Tilli M., Motooka T., Airaksinen V.M., Franssila S., Paulasto-Kröckel M., Lindroos V. (2015). Handbook of Silicon Based MEMS Materials and Technologies.

[30] McLean M.J., Osborn W.A. (2018). In-situ elastic strain mapping during micromechanical testing using EBSD. Ultramicroscopy.

[31] Prior D.J., Mariani E., Wheeler J. (2009). EBSD in the Earth Sciences: Applications, Common Practice, and Challenges. Electron Backscatter Diffraction in Materials Science.

[32] Wallis D., Hansen L.N., Britton T.B., Wilkinson A.J. (2019). High-Angular Resolution Electron Backscatter Diffraction as a New Tool for Mapping Lattice Distortion in Geological Minerals. J. Geophys. Res. Solid Earth.

[33] Humphreys F.J. (2001). Grain and subgrain characterisation by electron backscatter diffraction. J. Mater. Sci..

[34] Glez J.C., Driver J. (2001). Orientation distribution analysis in deformed grains. J. Appl. Crystallogr..

[35] Pantleon W. (2005). Retrieving orientation correlations in deformation structures from orientation maps. Mater. Sci. Technol..

[36] Nowell M.M., Wright S.I. (2017). Thoughts on Standards Materials and Analytical Routines for Electron Backscatter Diffraction (EBSD). Microsc. Microanal..

[37] Reimer L. (1998). Scanning Electron Microscopy—Physics of Image Formation and Microanalysis.

[38] Winkelmann A., Nolze G., Vespucci S., Naresh-Kumar G., Trager-Cowan C., Vilalta-Clemente A., Wilkinson A.J., Vos M. (2017). Diffraction effects and inelastic electron transport in angle-resolved microscopic imaging applications. J. Microsc..

[39] Hartley R., Zisserman A. (2003). Multiple View Geometry in Computer Vision.

[40] Oliphant T.E. (2006). A Guide to NumPy.

[41] Zhang Z. (2010). Estimating Projective Transformation Matrix (Collineation, Homography).

[42] Nolze G., Jürgens M., Olbricht J., Winkelmann A. (2018). Improving the precision of orientation measurements from technical materials via EBSD pattern matching. Acta Mater..

[43] Kirkland E.J. (2010). Advanced Computing in Electron Microscopy.

[44] Pan B., Xie H., Wang Z. (2010). Equivalence of digital image correlation criteria for pattern matching. Appl. Opt..

[45] Woodford O.J. (2018). Using Normalized Cross Correlation in Least Squares Optimizations. arXiv.

[46] Winkelmann A. (2018). xcdskd: Tools and Methods for Kikuchi Diffraction. https://github.com/wiai/xcdskd.

[47] Winkelmann A., Britton T.B., Nolze G. (2019). Constraints on the effective electron energy spectrum in backscatter Kikuchi diffraction. Phys. Rev. B.

[48] Johnson S.G. (2020). The NLopt Nonlinear-Optimization Package. https://github.com/stevengj/nlopt.

[49] Bachmann F., Hielscher R., Jupp P.E., Pantleon W., Schaeben H., Wegert E. (2010). Inferential statistics of electron backscatter diffraction data from within individual crystalline grains. J. Appl. Crystallogr..

[50] Wright S.I., Nowell M.M., Field D.P. (2011). A review of strain analysis using electron backscatter diffraction. Microsc. Microanal..

[51] MTEX (2020). Kernel Average Misorientation (KAM). https://github.com/mtex-toolbox/mtex.

[52] Wright S., Nowell M., Basinger J. (2011). Precision of EBSD based Orientation Measurements. Microsc. Microanal..

[53] Wright S.I., Basinger J.A., Nowell M.M. (2011). Angular Precision of Automated Electron Backscatter Diffraction Measurements. Mater. Sci. Forum.

[54] Thomsen K., Schmidt N.H., Bewick A., Larsen K., Goulden J. (2013). Improving the Accuracy of Orientation Measurements using EBSD. Microsc. Microanal..

[55] Nicolay A., Franchet J.M., Cormier J., Mansour H., Graef M.D., Seret A., Bozzolo N. (2018). Discrimination of dynamically and post-dynamically recrystallized grains based on EBSD data: Application to Inconel 718. J. Microsc..

[56] Barton N.R., Dawson P.R. (2001). On the spatial arrangement of lattice orientations in hot-rolled multiphase titanium. Model. Simul. Mater. Sci. Eng..

[57] Quey R., Driver J., Dawson P. (2015). Intra-grain orientation distributions in hot-deformed aluminium: Orientation dependence and relation to deformation mechanisms. J. Mech. Phys. Solids.

[58] Morawiec A. (2004). Orientations and Rotations.

[59] Albou A., Driver J.H., Maurice C. (2010). Microband evolution during large plastic strains of stable {110}〈112〉 Al and Al–Mn crystals. Acta Mater..

[60] Seret A., Moussa C., Bernacki M., Signorelli J., Bozzolo N. (2019). Estimation of geometrically necessary dislocation density from filtered EBSD data by a local linear adaptation of smoothing splines. J. Appl. Crystallogr..

[61] Morawiec A. (2003). A method of precise misorientation determination. J. Appl. Crystallogr..

[62] Kainuma Y. (1955). The theory of Kikuchi patterns. Acta Cryst..

[63] Michael J.R., Eades J.A. (2000). Use of reciprocal lattice layer spacing in electron backscatter diffraction pattern analysis. Ultramicroscopy.

[64] Winkelmann A. (2008). Dynamical effects of anisotropic inelastic scattering in electron backscatter diffraction. Ultramicroscopy.

[65] Bingham M.A., Nordman D.J., Vardeman S.B. (2010). Finite-sample investigation of likelihood and Bayes inference for the symmetric von Mises-Fisher distribution. Comput. Stat. Data Anal..

[66] Shi Q., Roux S., Latourte F., Hild F., Loisnard D., Brynaert N. (2018). On the use of SEM correlative tools for in situ mechanical tests. Ultramicroscopy.

[67] Ernould C., Beausir B., Fundenberger J.J., Taupin V., Bouzy E. (2020). Global DIC approach guided by a cross-correlation based initial guess for HR-EBSD and on-axis HR-TKD. Acta Mater..

[68] Szeliski R. (2011). Computer Vision.

